# Pretreatment of ovaries with collagenase before vitrification keeps the ovarian reserve by maintaining cell-cell adhesion integrity in ovarian follicles

**DOI:** 10.1038/s41598-020-63948-y

**Published:** 2020-04-22

**Authors:** Tomoko Kawai, Masayuki Shimada

**Affiliations:** Laboratory of Reproductive Endocrinology, Graduate School of Integrated Sciences for Life, Hiroshima University, Higashi-Hiroshima Hiroshima, Japan

**Keywords:** Biotechnology, Cell biology

## Abstract

The mammalian ovarian follicle is comprised of the germ cell or oocyte surrounded by the somatic cells, the granulosa and theca cells. The ovarian stroma, including the collagen-rich matrix that supports the three-dimensional disk-like follicular structure, impacts the integrity of the ovarian follicle and is essential for follicular development. Maintaining follicular integrity during cryopreservation has remained a limiting factor in preserving ovarian tissues for transplantation because a significant proportion of developed follicles in the frozen-thawed ovaries undergo atresia after transplantation. In this study, we show for the first time that during vitrification of the mouse ovary, the attachment of the oocyte to the granulosa cells was impaired by the loss of the cadherin adhesion molecules. Importantly, exposure to a high osmotic solution greatly decreased the ratio of oocyte diameter to the diameter of its follicle but did not alter the collagen-rich matrix surrounding the follicles. By treating ovaries briefly with collagenase before exposure to the hyper-osmotic solution the ratio of oocyte diameter to follicle diameter was maintained, and cadherin adhesion junctions were preserved. When frozen-thawed ovaries were transplanted to the bursa of recipient hosts, pretreatment with collagenase significantly increased serum levels of AMH, the number of intact follicles and the total number of viable offspring compared to frozen-thawed ovaries without collagenase pretreatment, even 6 months after transplantation. Thus, the collagenase pretreatment could provide a beneficial approach for maintaining the functions and viability of cryopreserved ovaries in other species and clinically relevant situations.

## Introduction

The fertility of young women with malignancies is dramatically decreased by chemical anti-cancer therapies^1,2^. To preserve patient fertility, assisted reproductive technology (ART) is often utilized. When ART is used in infertile women to obtain an increased number of mature oocytes that will enable the best probability of achieving a live birth, controlled ovarian stimulation (COS) is commonly used^3^. However, gonadotropin treatment can lead to the stimulation of non-physiological levels of estrogens that increase the risk of malignant progression in patients with the luminal type of breast cancer^4^. In the case of childhood cancer patients, most ovarian follicles are only at an early stage of development and lack the ability to respond to gonadotropins to reach the ovulatory stage^5^. Therefore, general ART procedures requiring mature oocytes are not adaptable for fertility preservation in young cancer patients. However, cryopreservation of ovarian tissue containing immature oocytes in preantral follicles is an experimental option for patients before the start of chemical anti-cancer therapy^6^.

A slow-freezing technique is commonly used for ovarian cryopreservation^7–9^. Ovarian tissues are treated with cryoprotectants such as DMSO, ethylene glycol or sucrose^10^. The ovarian tissues are cooled and then slowly frozen by a programmed freezer that decreases the temperature over a long period of time. The slow freezing process has been considered to be important for suppressing an increase in cell volume and ice crystal formation. However, Van der Ven *et al*.^11^ reported that among 49 women who received transplanted ovarian tissue after cryopreservation, the rate of pregnancy was only 33%. The limitation of the xenograft technique for delivering frozen-thawed human ovarian tissue to immunodeficient mice has also been reported; the morphology of the human frozen ovaries looks normal just after thawing, but a significant portion of the developed follicles undergo atresia within 6 days after *in vitro* culture^12^. The circulating levels of AMH secreted from small follicles are significantly lower in mice xenografted with human frozen-thawed ovaries compared with mice xenografted with fresh ovaries^13^.

The vitrification technique has been adapted for the cryopreservation of blastocyst embryos because the recovery rate of embryos after thawing is significantly higher than that of slow-frozen embryos^14–16^. The hyperosmotic vitrification solution removes the intercellular water before freezing, which results in a reduced risk of ice crystal formation in the cells during the freezing process. Therefore, vitrification is thought to be a universal technique for preserving tissues or cells. Apoptotic cells are not detected just after thawing of frozen-ovarian tissue frozen by vitrification^17^. However, after transplantation the function of frozen-thawed ovarian tissue preserved by vitrification did not improve compared to that of the slowly-frozen ovaries^18^. Furthermore, most follicles, except for primordial follicles, undergo atresia following transplantation or culture^19^.

Primordial follicles consist of an oocyte and one layer of flattened pregranulosa cells; importantly direct attachment of the oocyte to the pregranulosa cells is not observed at this stage^20,21^. However, direct communication between the oocyte and granulosa cells begins to occur as follicles leave the resting primordial pool to become primary follicles and plays an important role at all later stages of follicular development to ensure survival of the oocyte^22^. Developing follicles also contain a theca cell layer external to the basal lamina surrounding granulosa cells as well as stromal cells that produce a collagen-rich extracellular supportive matrix^23^. Thus, the maintenance of the spherical follicular structure is highly organized and requires precise attachment of each cell type within follicle and stroma. If the elegant follicular structure is disrupted by a hyperosmotic solution due to the different shrinking speeds among the cell types follicular integrity is compromised severely.

In this study, we focused on the integrity of cell-cell attachments within follicles and the morphological changes that occur in the ovarian stroma during the process of vitrification of the mouse ovary. Pretreatment of ovaries with collagenase maintained both the internal and external structures of developing follicles during exposure to a hyperosmotic solution and improved the reproductive performance of frozen-thawed ovaries after transplantation.

## Results

### A high osmotic vitrification solution damages cell adhesion between the oocyte and granulosa cells of growing ovarian follicles

**Figure 1:**
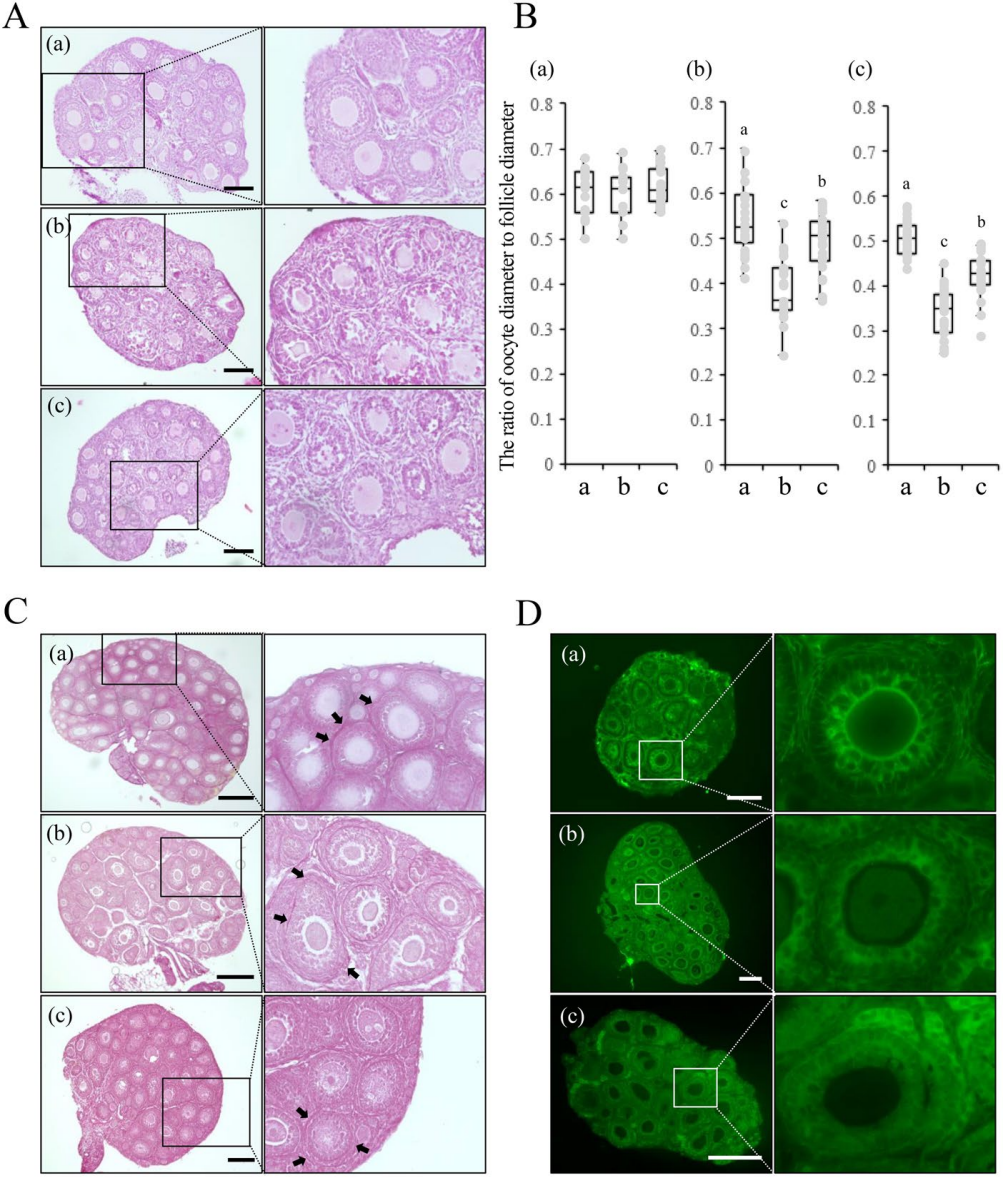
The high osmotic solution changes the morphology of the ovary during the vitrification process. (**A**) Image of HE-staining of mouse ovaries during the vitrification process. The scale bar is 100 μm. (a): An ovary collected from a 2-week-old mouse. (b): An ovary from a 2-week-old mouse treated with the high osmotic vitrification solution. (c): An ovary from a 2-week-old mouse treated with the normal osmotic solution following treatment with the high osmotic vitrification solution. (**B**) The ratio of oocyte diameter to follicle diameter of the ovaries of 2-week-old mice. From each ovary, 5 primary follicles and 5 secondary follicles were randomly selected, and the oocyte diameter and the follicle diameter were measured. The ratio of oocyte diameter to follicle diameter was calculated. In each treatment group, 5 ovaries were used for this calculation. In the box plot: bar = median, box = 25 th to 75 th percentiles, whiskers = 10 th and 90 th percentile. a; A fresh ovary. b; An ovary treated with the high osmotic vitrification solution. c; An ovary treated with the normal osmotic solution following treatment with the high osmotic vitrification solution. The different symbols represent significant differences (p < 0.05). (a): The ratio of oocyte diameter to follicle diameter of primary follicles. (b): The ratio of oocyte diameter to follicle diameter of secondary follicles with two layers of granulosa cells. The different symbols represent are significant differences. (c): The ratio of oocyte diameter to follicle diameter in secondary follicles with multilayered granulosa cells. The different symbols represent significant differences. (**C**) Image of PSR staining of mouse ovaries during the vitrification process. The scale bar is 100 μm. → indicates PSR positive layer. (a): An ovary collected from a 2-week-old mouse. (b): An ovary from a 2-week-old mouse treated with the high osmotic vitrification solution. (c): An ovary from a 2-week-old mouse treated with the normal osmotic solution following treatment with the high osmotic vitrification solution. (**D**) Pan-cadherin signal in a mouse ovary during the vitrification process. The scale bar is 100 μm. (a): An ovary collected from a 2-week-old mouse. (b): An ovary from a 2-week-old mouse treated with the high osmotic vitrification solution. (c): An ovary from a 2-week-old mouse treated with the normal osmotic solution following treatment with the high osmotic vitrification solution.

The ovaries of two-week-old mice contained primordial follicles, primary follicles and secondary follicles in which granulosa cells closely surrounded the oocytes (Fig. 1Aa). However, treatment with a high osmotic vitrification solution led to the formation of notable spaces/gaps between the oocyte and granulosa cells in secondary follicles, but not in primary and primordial follicles (Fig. 1Ab,c). The ratio of oocyte diameter to follicle diameter was similar in primary follicles before and after the treatment (Fig. 1Ba). However, this ratio was significantly decreased in secondary follicles exposed to similar treatments (Fig. 1Bb). Although the higher ratio was partially restored after the exposure to a normal osmotic solution, the ratio was still significantly lower than that in untreated ovaries (Fig. 1Bc).

To understand the underlying mechanisms by which the ratio of oocyte diameter to follicle diameter in secondary follicles was reduced by treatment with the high osmotic vitrification solution, the structural organization of the secondary follicles was examined by visualizing collagen with Picrosirius Red (PSR) and junctional complexes by immunostaining for the cell adhesion molecules pan-cadherins. The PSR positive collagen layer was localized to the boundary between each ovarian primary or secondary follicle and the ovarian stroma but not between the primordial follicle and the ovarian stroma (Fig. 1Ca). After treatment with the high osmotic vitrification solution and/or following exposure to the normal osmotic solution, the boundaries were still observed upon the PSR positive layer in primary and secondary follicles (Fig. 1Cb,c).

The attachment of the oocyte to granulosa cells and a strong cadherin signal between these cells were detected in secondary follicles but not in primary follicles in fresh ovaries (Fig. 1Da). Cadherin signals in secondary follicles disappeared after treatment with the high osmotic vitrification solution (Fig. 1Db). Cell-cell adhesion, mediated by cadherin, was not restored after the exposure to the normal osmotic solution (Fig. 1Dc).

**Figure 2:**
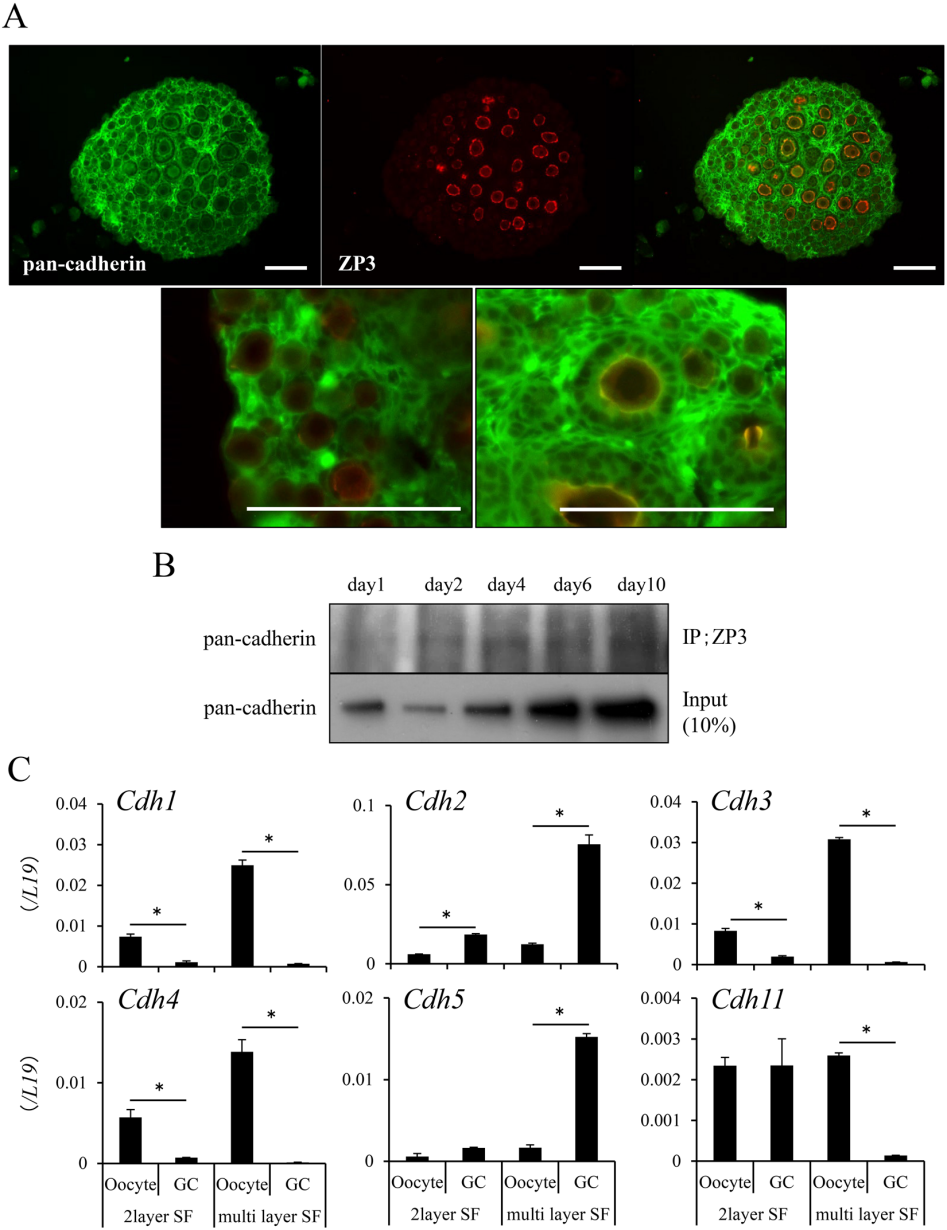
Changes in the expression and localization of cadherin in mouse ovaries during the follicular development process. (**A**) The localization of cadherin in 1-week-old mouse ovaries. Cadherin was visualized by the green fluorescence signal and the zona pellucida was identified with ZP3 (red signal). The scale bar is 100 μm. (**B**) Kinetic changes in the coexpression of ZP3/cadherin in mouse ovaries. Protein samples from mouse ovaries were incubated with an anti-ZP3 antibody. Proteins immunoprecipitated with an anti-ZP3 antibody were loaded and then detected by an anti-pan-cadherin antibody. Ten percent of the protein samples purified from ovaries were used as loading controls. The results are representative of three independent experiments. (**C**) The expression of each cadherin family member in oocytes or granulosa cells in secondary follicles with two layers of granulosa cells or secondary follicles with multilayered granulosa cells. * denotes a significant difference was between oocyte and GCs (p < 0.05). 2layer SF: secondary follicle with two layers of granulosa cells, multi-layer SF: secondary follicle with multilayered granulosa cells, GC: granulosa cells. The expression levels of genes were normalized according to that of *L19*.

Cell-cell adhesions between the oocyte and granulosa cells were formed both by cadherins and the zona pellucida protein 3 (ZP3). Such complexes appeared in secondary follicles but not in primary follicles. Both cadherin expression and complex formation with ZP3 were significantly increased in ovaries of mice from 1 day to 10 days of age. Some of the genes encoding members of the cadherin family were dominantly expressed in oocytes in secondary follicles. Others were expressed in granulosa cells, and both cadherins and ZP3 were more strongly induced in developed secondary follicles than in 2-layer secondary follicles (Fig. 2).

### Pretreatment with collagenase maintained the cell-cell contacts between the oocyte and granulosa cells in secondarily follicles

**Figure 3:**
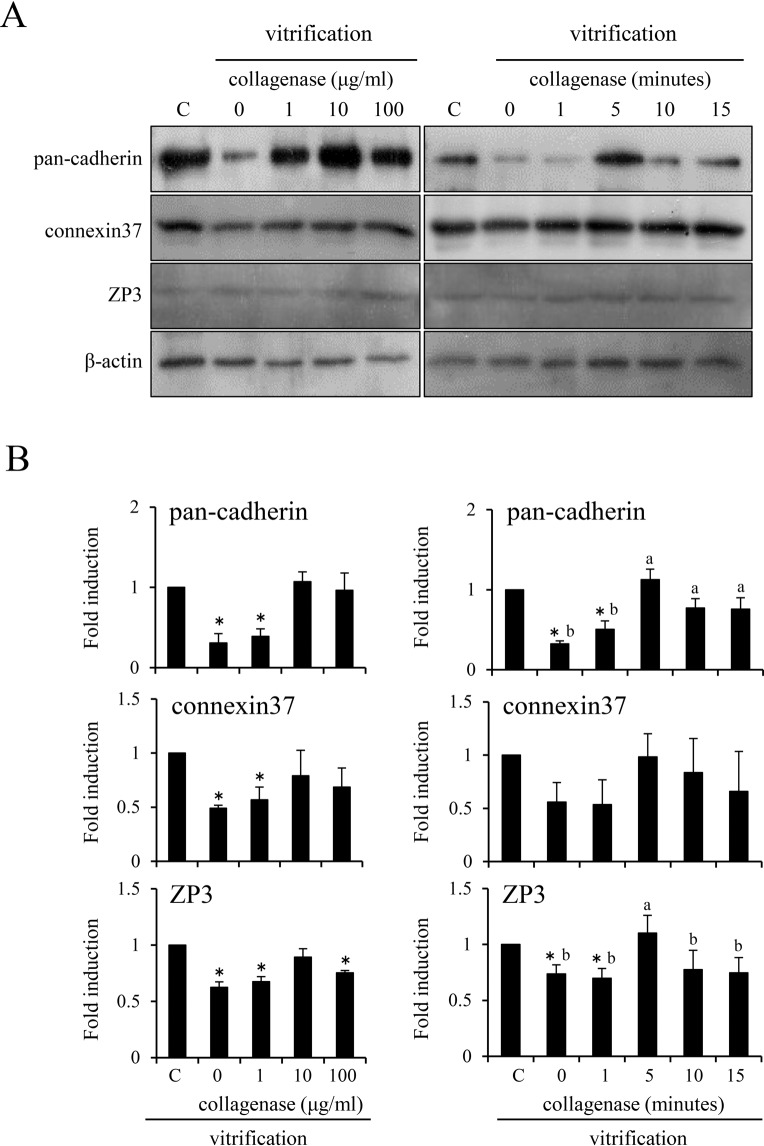
Effects of collagenase pretreatment on the expression of adhesion proteins in frozen-thawed mouse ovaries. (**A**) The dose-dependent effects of collagenase pretreatment (0, 1, 10, 100 μg/ml) and the time-dependent effects of pretreatment with 10 μg/ml collagenase before exposure to the high osmotic solution. Two-week-old mouse ovaries were treated with 0, 1, 10 or 100 μg/ml collagenase for 5 min and underwent the vitrification process. Frozen-ovaries were thawed and then used for western blotting analysis. β-actin was used as a loading control. The results are representative of three independent experiments. C; Two-week-old mouse fresh ovaries. (**B**) Quantitative expression of pan-cadherin, connexin37 and ZP3 relative to the expression of β-actin (control), as determined by western blotting. The control value was set as 1, and the data are expressed as fold induction. The values are the mean ± SEM of three replicates. * denotes significant differences compared with the control (p < 0.05). Different superscripts denote significant differences for the different durations of treatment with 10 μg/ml of collagenase.

The size of the oocyte but not that of the follicle was reduced by the high osmotic solution; however, the collagen-basement membrane was maintained in each follicle during the vitrification process. Therefore, the effects of pretreatment with collagenase on the subsequent exposure to the high osmotic solution were examined. The levels of pan-cadherin, connexin37 (Cx37) and ZP3 protein in the ovaries were significantly decreased by treatment with the high osmotic solution. However, no reduction of either pan-cadherin or Cx37 was observed when the ovaries were pretreated with 10 or 100 μg/ml collagenase for 5 min. The level of ZP3 was restored at 10 μg/ml, however, the level was significantly decreased by pretreatment with 100 μg/ml collagenase. Longer-term treatment with 10 μg/ml of collagenase (10 or 15 min) also significantly decreased the level of cadherin and ZP3 in the ovaries (Fig. 3A,B). Therefore, we used 10 μg/ml collagenase for 5 min as the optimal conditions of treatment in subsequent experiments.

**Figure 4:**
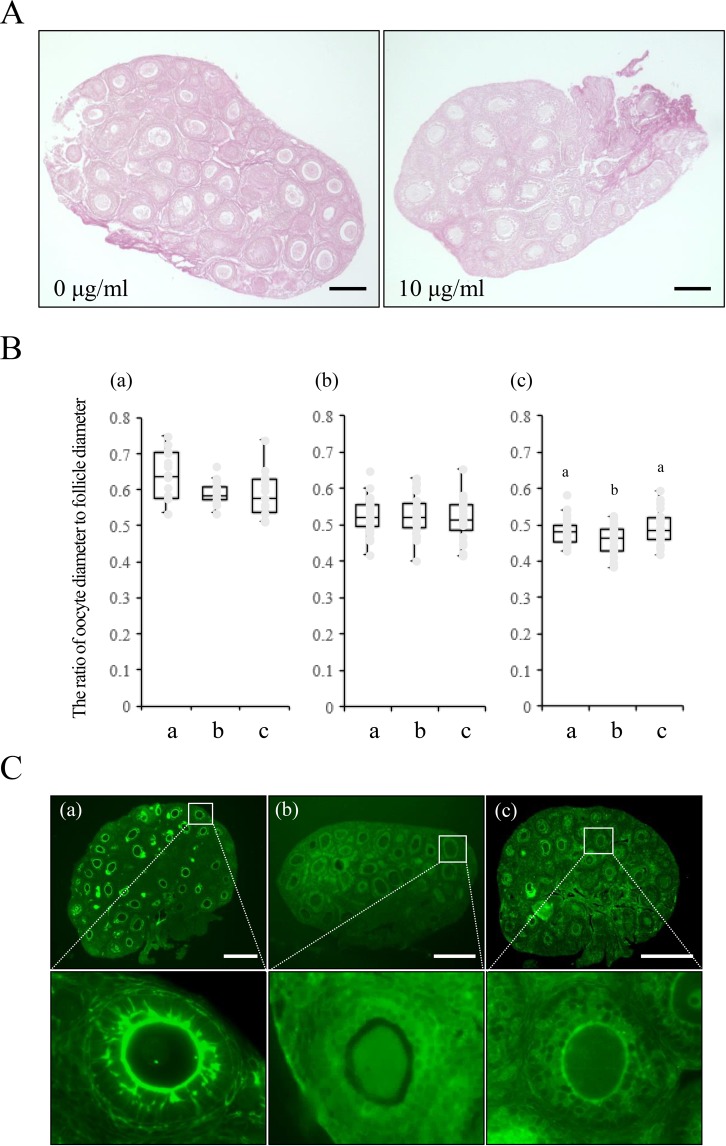
Effect of collagenase pretreatment on the vitrification of mouse ovaries. (**A**) The ovaries of mice treated with or without collagenase were stained with PSR to detect the localization of collagen. The scale bar is 100 μm. (**B**) The ratio of oocyte diameter to its follicle diameter of ovaries treated with collagenase before exposure to the high osmotic solution. For each ovary, 5 primary follicles and 5 secondary follicles were randomly selected, and the oocyte diameter and follicle diameter were measured by ImageJ software. The ratio of oocyte diameter to follicle diameter was calculated. In each treatment group, 5 ovaries were used for this calculation. In the box plot: bar = median, box = 25 th to 75 th percentiles, whiskers = 10 th and 90 th percentile. a; A fresh ovary. b; An ovary treated with high osmotic vitrification solution. c; An ovary treated with the normal osmotic solution following treatment with the high osmotic vitrification solution. The different symbols represent significant differences. The different symbols represent significant differences (p < 0.05). (a): The ratio of oocyte diameter to follicle diameter of primary follicles. (b): The ratio of oocyte diameter to follicle diameter of secondary follicles with two layers of granulosa cells. The different symbols represent significant differences. (c): The ratio of oocyte diameter to follicle diameter of secondary follicles with multilayered granulosa cells. The different symbols represent significant differences. (**C**) The effects of 10 μg/ml collagenase pretreatment for 5 min on the expression of pan-cadherin in frozen-thawed mouse ovaries. The scale bar is 100 μm. (a): The localization of cadherin in ovaries from 2-week-old mice. (b): Frozen-thawed ovaries from 2-week-old mice without collagenase pretreatment. (c): Frozen-thawed ovaries from 2-week-old mice with collagenase pretreatment.

Under the optimal conditions of collagenase treatment, (1) cadherin signals were strongly detected in the matrix between the oocyte and granulosa cells in secondary follicles of ovaries that were exposed to the high osmotic solution (Fig. 4A,C); (2) the ratio of oocyte diameter to follicle diameter was not significantly changed (Fig. 4B) and (3) the ZP3 and cadherin complex was still detected.

**Figure 5:**
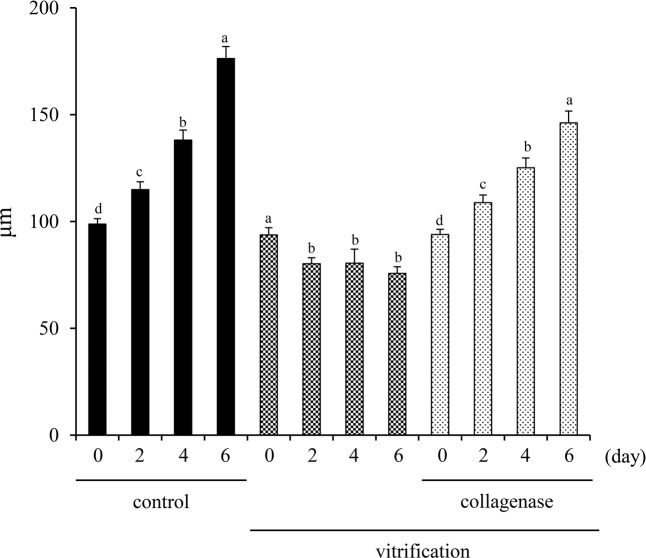
Effect of collagenase pretreatment on the development of secondary follicles collected from frozen-thawed ovaries. After thawing, secondary follicles with multilayered granulosa cells collected and then were cultured for 6 days in culture medium. The diameter of each secondary follicle was measured every 2 days. * denotes a significant difference between day 0 and each time point in each treatment group (p < 0.05).

To examine the developmental competence of frozen-thawed secondary follicles after pretreatment with collagenase, multilayered secondary follicles were collected from fresh or frozen-thawed ovaries and then cultured with FSH and serum. When secondary follicles were collected from fresh ovaries, the diameter of each secondary follicle was significantly increased in a time-dependent manner in culture. An increase in diameter was not observed in secondary follicles collected from frozen-thawed ovaries without collagenase pretreatment but did increase in secondary follicles collected from frozen-thawed ovaries pretreated with collagenase and in a time-dependent manner similar to that of secondary follicles collected from fresh ovaries (Fig. 5).

### Pretreatment with collagenase maintained ovarian function when frozen-thawed ovaries were transplanted to female host mice

To determine whether frozen-thawed ovaries pretreated with or without collagenase performed normal ovarian functions, the ovaries of 25-day-old mice were cryopreserved by vitrification with or without pretreatment with collagenase. The frozen-thawed ovaries were then transferred to the bursae in an adult female mouse from which the ovaries had been removed.

**Figure 6:**
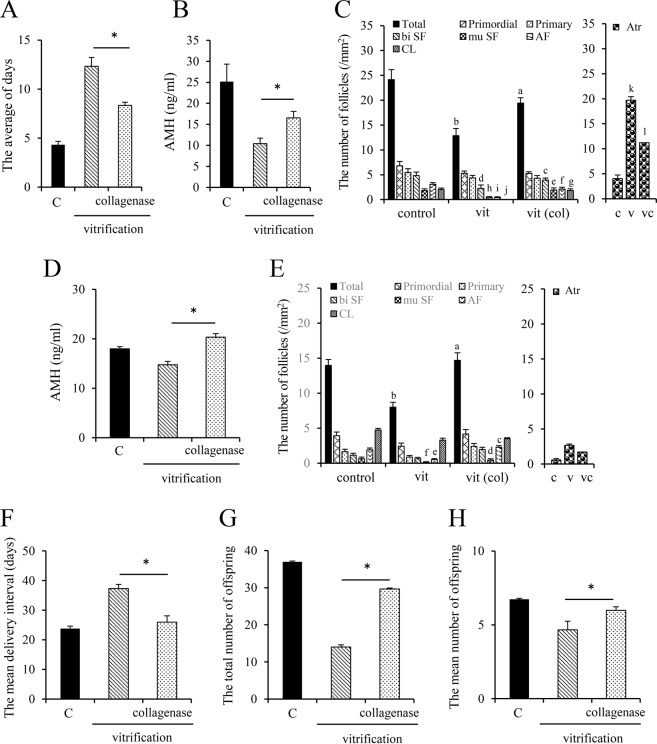
The function of 25-day-old mouse ovaries cryopreserved after collagenase pretreatment. (**A**) The average number of days required for the recovery of the estrus stage in recipient adult female mice transplanted with the frozen-thawed ovaries. The ovaries of 25-day-old mice were frozen by vitrification with or without collagenase treatment. The ovaries were thawed with culture medium and replaced with both ovaries of another adult female mouse. Beginning the day following ovarian transplantation, vaginal smear tests were performed to examine which stage of ovarian follicle was preserved in the ovary. * denotes a significant difference between the treatment groups (p < 0.05). (**B**) The circulating level of AMH 1 week after ovarian transplantation. * denotes a significant difference between the treatment groups (p < 0.05). (**C**) The number of follicles at each stage in each ovary 1 week after transplantation. The different symbols denote significant differences between vitrificated ovaries (vit) and vitrificated ovaries pretreated with collagenase [vit (col)] (p < 0.05). (**D**) The circulating level of AMH 6 months after ovarian transplantation. * denotes a significant difference between the treatment groups (p < 0.05). (**E**) The number of follicles at each stage in each ovary 6 months after transplantation. The different symbols denote significant differences between vitrificated ovaries (vit) and vitrificated ovaries pretreated with collagenase [vit (col)] (p < 0.05). (**F**) The mean delivery interval of recipient adult female mice 6 months after frozen-thawed ovaries were transplanted. * denotes a significant difference between the treatment groups (p < 0.05). (**G**) The total number of offspring of recipient adult female mice 6 months after frozen-thawed ovaries were transplanted. * denotes a significant difference between the treatment groups (p < 0.05). (**H**) The mean number of offspring of recipient adult female 6 months after frozen-thawed ovaries were transplanted. * denotes a significant difference between the treatment groups (p < 0.05).

The average number of days it took for the mice to return to the estrous stage was (1) approximately 4 days when fresh ovaries were transferred to host female mice (controls); (2) more than 13 days when frozen-thawed ovaries not pretreated with collagenase were transferred to female host mice and (3) approximately 8 days for ovaries pretreated with collagenase (Fig. 6A). Serum levels of AMH in mice 1 week after the transplantation of frozen-thawed ovaries were significantly lower in non-collagenase treated ovaries than (1) those in control mice (transplanted with fresh ovaries) and (2) in collagenase-pretreated ovaries (Fig. 6B). The total number of non-atretic follicles with a clear basement membrane was significantly higher 1 week after transplantation in the collagenase pretreatment group than in the group transplanted with frozen-thawed ovaries without collagenase treatment (Fig. 6C). However, there was no significant difference in the number of primordial follicles or primary follicles among the treatment groups (Fig. 6C). Compared with ovary without the pretreatment, the pretreatment with collagenase before vitrification significantly increased the number of developed follicles from bilayer secondary follicles (Fig. 6C).

Six months after the transplantation of frozen-thawed ovaries, serum levels of AMH were significantly higher in the collagenase-pretreated group than in the group without collagenase pretreatment (Fig. 6D). The total number of non-atretic follicles was also significantly higher in the collagenase-pretreated group (Fig. 6E). Pretreatment with collagenase also (1) shortened the mean delivery interval to approximately 26 days compared to approximately 37 days in mice transplanted with frozen-thawed ovaries without collagenase pretreatment (Figs. 6F) and (2) significantly increased the total number of offspring born 6 months after transplantation and the mean number of offspring per mouse (Fig. 6G,H). These positive effects of collagenase pretreatment on AMH levels, the number of healthy follicles and fertility were also observed when ovaries from mice collected at 3-months age were used for vitrification (Supplemental Fig. S3).

## Discussion

The physical fusion/attachment of the granulosa cell membrane projections with those of the oocyte is observed in secondary follicles^24^, indicating that this specific contact communication mechanism between the oocyte to granulosa cells is essential for early stages of follicular development. Therefore, the preservation of these junctions during cryopreservation of ovarian tissues is also likely to be critical for successful ovarian transplantation and fertility restoration in cancer patients. Furthermore, the ovarian stromal tissue compartment that surrounds each follicle also regulates and maintains ovarian follicular structure. In particular the presence of stromal collagen impacts the rigidity of the tissue surrounding each follicle and this in turn impacts follicular function and development involving the maintenance of cell-cell communication between the oocyte and the granulosa cells.

The studies presented herein document that the rigidity of the stromal collagen impacts the ability of follicles to accommodate and survive the current cryopreservation processes and recover once the tissues are transplanted in culture and *in vivo*, allowing follicular development to proceed and restore fertility. More specifically, the maintenance of the cell-cell communication between the oocyte and the granulosa cells that begins at the secondary follicle stage is essential for recovery following cryopreservation and depends on protecting the integrity of specific junctional complexes between the oocyte and granulosa cells. These complexes are comprised of the zona pellucida, gap junctions and adherens junctions.

The formation of junctional complexes between the oocyte and granulosa cells begins during the transition of primordial follicles to primary-secondary follicles when the zona pellucida (ZP) glycoproteins are secreted and form a matrix between oocyte and granulosa cells^25^. Although a perivitelline space appears between the ZP and the oocyte cell membrane^26,27^, transzonal projections (TZPs) form between the oocyte and granulosa cells and are physiologically and functionally critical for follicular survival^28,29^. One of the components of TZPs is connexon, a hexamer of connexin37 (Gja4), which restricts gap junctional communication transport to factors with a molecular weight lower than 1,000 Da^30^. *Gja4*-deficient mice exhibit infertility due to failure of follicular development^22^. Cadherins are additional well-known cell-cell adhesion factors that form adherens junctions, usually found in epithelial cell junctional complexes and are dependent on calcium ions^31^. When mouse ovaries are cultured with the calcium chelator, EGTA, the structure of primordial follicles is not affected by the treatment, however the contact between granulosa cells and the oocyte is impaired by EGTA in secondary follicles^32^. As shown in this study, pan cadherin signals were strongly observed in the space between oocyte and granulosa cells and these signals were co-localized with ZP3 protein in secondary follicles. Therefore, both communicating junctions via Gja4 and anchoring junctions via cadherin are formed between the oocyte and granulosa cells in secondary follicles and impact the maintenance of their structure.

We document in these studies that improved cryopreservation techniques, specifically the use of a brief, low dose collagenase treatment, improve fertility outcomes in mice. The underlying basis for the improved preservation is related to accommodating high osmotic solutions. By exposing mouse ovaries to a high osmotic solution during the vitrification process, the contacts between granulosa cells and the oocyte in secondary follicles were lost as shown by the decrease in the pan cadherin signal. The space between the oocyte and granulosa cell layer was reduced after the ovary was thawed; however, cadherin did not return to the adhesive junction. Secondary follicles isolated from frozen-thawed ovaries or fresh ovaries were cultured to evaluate their developmental competence. The diameter of secondary follicles was increased in a time-dependent manner when follicles were isolated from fresh ovaries. However, this effect was completely suppressed in secondary follicles from frozen-thawed ovaries. It is known that cadherins play an important role in membrane fusion, including endocytosis^33^, exocytosis^34^ and cell-cell membrane fusion^35^. Oocyte-secreted factors, such as GDF9 and BMP15, are released from oocytes via the exocytosis system and then act on granulosa cells to induce cell proliferation^36^. Komatsu and Masubuchi (2018)^30^ reported that cell-cell membrane fusion works as a transporter system to transport molecules with a molecular weight greater than 10,000 Da between granulosa cells and oocyte. Such molecules are too large for transport via gap junctional communication^37^, suggesting that the presence of cadherins as well as gap junctions linking the oocyte and granulosa cells are critical for the transport of factors to promote follicular development.

To determine a possible mechanism by which cadherin adhesion between the oocyte and granulosa cells was lost by traditional vitrification procedures, we focused on the stromal collagen-rich matrix surrounding each follicle. Collagen fibers connect to form and create a cell-matrix tension^38–40^. Because exposure to the high osmotic solution during the vitrification process decreases the cell volume^41^ and because the size of oocytes was dramatically reduced by the high osmotic solution, we hypothesized that the granulosa cell layers are pulled by the collagen-rich matrix to create a space between the granulosa cells and the oocyte. Strikingly, a brief treatment with collagenase before exposure to the hyper osmotic solution maintained the cadherin adhesions between the oocyte and granulosa cell layers. As a consequence, secondary follicles collected from frozen-thawed ovaries that were pretreated with collagenase showed normal follicular development. Vitrification of ovaries is known to promote an atretic process in most follicles, except for primordial follicles^19^. However, pretreatment with collagenase prevents atresia and enables the maintenance of developmental competence of secondary follicles.

Follicular growth in culture is enhanced and maintained with appropriate amounts of either collagen or alginate providing evidence that the rigidity of the surrounding matrix impacts follicle growth and function. Our studies support these observations by showing that the dose and the duration of collagenase treatment of ovaries prior to vitrification is important for follicular integrity and function in frozen-thawed ovaries. Both a high dose and a long duration of collagenase treatment decreased the level of cadherin in ovaries, however, the optimal condition of collagenase pretreatment (10 μg/ml for 5 min) maintained healthy follicles after transplantation. Surprisingly, serum levels of AMH in mice transplanted with collagenase-treated cryopreserved ovaries was similar to that in control mice after 6 months. AMH is a well-known marker of the ovarian reserve that reflects both the number and quality of follicles in the ovary, especially from the primary to secondary stages where AMH is highest^42^. In this study, the number of primordial follicles did not significantly change by the pretreatment with collagenase, whereas the total number of ovarian follicles, especially secondary follicles, that survived were significantly increased at 6 months in the frozen-thawed, transplanted ovaries. Moreover, we have reported that in mice containing an abnormal ovarian stroma, the proliferation of granulosa cells of secondary follicles is impaired^43^. Therefore, increasing both the level of AMH and the number of developed follicles after 6-months suggests that the pretreatment with collagenase not only improved the quality of secondary follicles directly during the freezing process but also affected the functions of the surrounding ovarian stroma to support follicular development during 6 months after transplantation. Additionally, compared with the conventional vitrification methods, collagenase pretreatment increased the reproductive performance of mice with transplanted ovaries; the mean number of offspring was 5.98 vs. 4.67 and the total litter size 6 months after transplantation was 29.67 vs. 14.00, and the average days of delivery interval was 25.96 days vs. 39.33 days, respectively. These results are substantially better than those reported by Hani *et al*.^44^ in which vitrified ovaries from 4-week-old GFP-labeled mice were transplanted into the ovarian bursae on one side of 10-week-old non-GFP-labeled mice; the resulting percentage of GFP-positive pups 2 months after the mating test was only 16%.

Thus, our vitrification procedure using pretreatment of mouse ovaries with collagenase offers a new vitrification technology that strikingly maintains not only secondary follicle viability but also fertility in transplanted ovaries. However, to adapt this technology to other species the amount of collagenase used, and the duration of treatment will need to be adjusted because the characteristics of the ovarian stroma and the extracellular matrix (ECM) that surrounds the ovarian follicle differ among species^45^. For example, the thickness of the ovarian stroma in mice is approximately 2–3 mm and in 40-day-old mouse ovaries, approximately 5,000 follicles are present^46^. On the other hand, the diameter of human ovary is about 30–50 mm and there are about 50,000 follicles in the ovaries of 30-year-old women^47,48^. Thus, the number of follicles in human ovaries is less than one hundredth that of follicles in mouse ovaries; the human ovarian cortex is thick, and a developed collagen-rich matrix surrounds the follicle at each stage. Therefore, during conventional vitrification of human ovaries, approximately 1 mm of ovarian cortex tissue is cut from the surface of the ovary to increase the penetration of the high osmotic solution^49^. Based on results in the present study, an additional precollagenase step before exposure to the high osmotic extender could have been adjusted for the collagen-rich matrix-based cortex. Moreover, the composition of the ovarian stroma changes during the estrous cycle/ menstrual cycle and is altered as ovaries age in both mice and human^50^. In this study, the pretreatment of collagenase was adapted to the adult mice ovary. Because fibrosis with the accumulation of collagenase is increased in the ovarian stroma of the aged mice ovary^50^, a higher dose and the longer exposure time of collagenase would be required for an aged ovary.

In summary, using the appearance of a space between oocyte and granulosa cells, the maintenance of cadherin expression for functional attachment of the oocyte and granulosa cells and serum levels of AMH as an index of the follicular reserve, these criteria can be used to determine the optimal condition of collagenase pretreatment that supports follicular integrity. This novel approach for preserving follicular structure and function during vitrification could be potentially useful for many situations of clinical relevance.

## Methods

### Materials

DMEM/F12 and penicillin-streptomycin were purchased from Invitrogen (Carlsbad, CA, USA), fetal bovine serum (FBS) was purchased from Life Technologies Inc. (Grand Island, NY, USA), oligonucleotide poly-(dT) was purchased from Invitrogen, and AMV reverse transcriptase was purchased from Promega Corp (Promega; Madison, WI, USA). Routine chemicals and reagents were obtained from Nacalai Chemical Co. (Osaka, Japan), or Sigma Chemical Co. (St. Louis, MO, USA).

### Animals

C57BL/6 mice were obtained from Charles River Laboratories Japan (Yokohama, Japan). The animals were housed under a 12-h light/12-h dark schedule in the Experimental Animal Center at Hiroshima University and provided with food and water ad libitum. The animal study was approved by the Hiroshima University Animal Committee (Permit Number: C18–34), and the mice were maintained in accordance with the Hiroshima University Guidelines for the Care and Use of Laboratory Animals.

### Cryopreservation by vitrification

Ovarian cryopreservation was performed using Ova Cryo Kit Type M (Kitazato, Shizuoka, Japan)^49^. Whole ovaries were collected from 10-day-old, 14-day-old, 25-day-old or 3-month-old mice. Two of the ovaries were added to 100 μl Cryo1 and then kept for 5 min at room temperature. The ovaries were moved to fresh 1.5 ml of tube containing 100 μl of Cryo2 and the kept for 5 min. The ovaries were further transferred to fresh tube containing 100 μl of Cryo3 and then kept for 15 min at room temperature. After the treatment with Cryo3, the ovaries with a few volumes of cryo3 were filled into 0.5 ml plastic straw (Fujihira, Tokyo, Japan) without sealing and then placed in liquid nitrogen. For thawing, the plastic straw was quickly transferred from liquid nitrogen to 1.5 tube containing 1 ml of thawing solution (Thaw1) at 37 °C and gently shacked for 1 min to move ovaries from straw to the solution. The ovaries were moved to the fresh tube containing 1 ml of Thaw2 and then kept for 3 min. The ovaries were further transferred to 1 ml of Thaw3 and incubated for 5 min at 37 °C.

### Pretreatment with collagenase before vitrification

Ovaries were treated with 0, 1, 10 or 100 μg/ml collagenase (Wako, Osaka, Japan, 038–22361) solution, dissolved in culture medium (DMEM/F12) before treatment with Cryo1 at room temperature for 1, 5, 10 or 15 min.

### Ovarian transplantation

Adult (8-week-old) female C57BL/6 mice were anesthetized with pentobarbital (Kyoritsu Seiyaku, Tokyo, Japan) and isoflurane (Pfizer, NY, USA). Both ovaries were removed surgically from the bursa surrounding the ovaries and each was replaced with frozen-thawed ovaries. The skin was sutured after the operation.

### Vaginal smear tests

Vaginal smear tests were done according to Kawai *et al*.^51^. Vaginal components were collected in PBS (-) at the same time each morning and were smeared onto a glass slide and fully air dried. These samples were fixed with methanol (Kishida Chemical Co, Japan). After fixation, these samples were washed with water from the back side of the glass slide and stained with Giemsa stain (Wako). Vaginal smears were observed under a CX41 microscope (Olympus, Tokyo, Japan).

### Mating test

Mating experiments were carried out using 3 females from each treatment group beginning 1 month after ovarian transplantation. Adult male mice were placed in each cage for 6 months, and the number of pups in each litter and the days of pregnancy were recorded.

### Isolation and *in vitro* culture of secondary follicle

Frozen-thawed ovaries were moved to 35 mm dish (SUMILON, Tokyo, Japan) containing 3 ml of DMEM/F12 medium. Secondary follicles were isolated from frozen-thawed ovaries by the puncture with 26G 1/2 needle (Terumo corporation) under the stereo microscope. The secondary follicles were selected by glass pipette. After washing with PBS (-), the secondary follicles were placed in 0.5% (w/v) sodium alginate (Wako)/PBS (Ca^2+^-and- Mg^2+^ -free) and then placed in 50 mM CaCl_2_/saline to cross-link the alginate. Ten of secondary follicles were placed in one alginate bead and were cultured for 6 days in the medium (DMEM/F12 containing penicillin and streptomycin) containing 10 ng/ml ovine FSH (NIDDK, Torrance, CA, USA) and ITS Supplement (Sigma) in the presence of 1% (v/v) FBS. The culture medium was changed every 2 days and these secondary follicles were captured using a Keyence BZ-9000 microscope (Keyence Co., Osaka, Japan). The area of each secondary follicle was measured by a BZ analyzer.

### RNA extraction and real-time PCR

RNA extraction and real-time PCR were done according to Shimada *et al*.^52^. Total RNA was obtained from mouse granulosa cells from 50 secondary follicles using an RNAeasy Mini Kit (Qiagen Sciences, Germantown, MD, USA) according to the manufacturer’s instructions. Fifty micrograms of total RNA were reverse transcribed using 500 ng poly-dT (Invitrogen) and 0.25 U avian myeloblastosis virus-reverse transcriptase at 42 °C for 75 min and 95 °C for 5 min. cDNA and primers were added to 15 μl total reaction volume of Power SYBR Green PCR Master Mix (Applied Biosystems, Foster City, CA, USA). PCR was then performed using the StepOne real-time PCR system (Applied Biosystems). The conditions were set to the following parameters: 10 min at 95 °C followed by 40 cycles of 15 seconds at 95 °C and 1 min at 60 °C. The primer sets are shown in Table S1. *L19* was used as a control for reaction efficiency and variations in the concentrations of mRNA in the original RT reaction.

### Western blotting

Western blotting was done according to Shimada *et al*.^53^. Protein samples were obtained by homogenizing secondary follicles and lysing them in RIPA lysis buffer (Santa Cruz Biotechnology Inc, TX, USA) and then diluted with 2× SDS sample buffer. Proteins (20 μg) were separated by SDS polyacrylamide gel (10%) electrophoresis and transferred to PVDF membranes (GE Bioscience, USA). The Membranes were blocked in Tris-buffered saline and Tween 20 (TBST; 10 mM Tris (pH 7.5), 150 mM NaCl and 0.05% Tween 20) containing 5% BSA (Nacalai Chemical Co.). The blots were incubated with primary antibody (1:500 anti-pan cadherin, Biosensis Australia; 1:500 anti-connexin37, Abcam; 1:500 anti-ZP3, Proteintech, Chicago, USA; 1:5000 anti-β-actin, Sigma) overnight at 4 °C. After washing in TBST, enhanced chemiluminescence (ECL) detection was performed by using the ECL system according to the manufacturer’s specifications (GE Bioscience) and appropriately exposing the blots to Fuji X-ray film (Fujifilm, Tokyo, Japan). The expression of each protein was calculated by the Gel Analyzer of ImageJ.

### Immunoprecipitation

Secondary follicles or ovaries (from 1, 2, 4, 6 or 10-day-old mice) were lysed with RIPA lysis buffer (Santa Cruz Biotechnology Inc). Proteins (150 μg) were treated with primary antibody (1:50 anti-ZP3, Proteintech) overnight at 4 °C. The mixture was further incubated with 10 µl of Protein G Magnetic Beads (Cell Signaling Technology, Tokyo, Japan) for 30 min at 4 °C and then washed 5 times in RIPA lysate buffer. The precipitates were suspended in 15 μl of 2 × SDS sample buffer. These were separated by SDS polyacrylamide gel (10% (v/v)) electrophoresis and transferred to PVDF membranes (GE Bioscience). The membranes were blocked in Tris-buffered saline and Tween 20 (TBST; 10 mM Tris (pH 7.5), 150 mM NaCl and 0.05% Tween 20) containing 5% (w/v) BSA (Nacalai Chemical Co.). The blots were incubated with primary antibody (1:500 anti-pan cadherin) overnight at 4 °C. After washing in TBST, enhanced chemiluminescence (ECL) detection was performed by using the ECL system according to the manufacturer’s specifications (GE Bioscience) and appropriately exposing the blots to Fuji X-ray film (Fujifilm).

### Immunofluorescence staining

Immunofluorescence staining was done according to Kawai *et al*.^51^. Ovaries were fixed in 4% paraformaldehyde (Nacalai Chemical Co.) for 24 h at 4 °C. Subsequently, these tissues were washed with PBS and embedded in paraffin wax (Thermo Fisher Scientific Inc., Waltham, USA) after dehydrated. Paraffin-embedded tissue sections (5 μm) were deparaffinized. After washing with PBS, the sections were treated with 10 mM citric acid buffer (pH 6.0) (Nacalai Chemical Co.) for 20 min in boiling water for antigen activation. Then, the sections were washed 3 times for 5 min with PBS (-) and blocked with 5% (v/v) BSA (Nacalai Chemical Co.). The sections were incubated with primary antibody (1:100 anti-ZP3, Proteintech) in 0.3% (v/v) Triton X-100 in PBS (-) containing 5% (v/v) BSA (Nacalai Chemical Co.) overnight at 4 °C. overnight at 4 °C. After washing with 0.3% (v/v) Triton X-100 in PBS (-), the ovaries were visualized with an Alexa Fluor 568-conjugated goat anti-rabbit secondary antibody (1:100) (Sigma) and DAPI (Vector Laboratories Inc., CA, USA). For pan-cadherin staining, after washing with PBS (-), the sections were blocked with the M.O.M kit (VECTOR) as a mouse IgG blocking reagent to reduce the endogenous mouse IgG staining when using mouse primary antibodies on mouse tissue according to manufacturer protocol. The sections were incubated with primary antibody (1:100 anti-pan cadherin) in 0.3% (v/v) Triton X-100 in PBS (-) containing mouse IgG blocking reagent overnight at 4 °C. After washing with 0.3% (v/v) Triton X-100 in PBS (-), the ovaries were visualized with an Alexa Fluor 488-conjugated anti-mouse secondary antibody (1:100) (Sigma) and DAPI (Vector Laboratories Inc., CA, USA). Digital images were captured using a Keyence BZ-9000 microscope (Keyence Co).

### Hematoxylin and eosin staining

Ovaries were fixed, embedded and sectioned as described above. After deparaffinization and washing with PBS (-), the tissues were stained with eosin (Sakura-Finetek Japan, Osaka, Japan) and hematoxylin (Sakura-Finetek Japan) to visualize the cytoplasm and nuclei, respectively. Tissues were observed under a Keyence BZ-9000 microscope. For calculating the ratio of oocyte diameter to follicle diameter, 5 primary follicles and 5 secondary follicles were randomly selected in each ovary, and the oocyte diameter and the follicle diameter in each follicle were measured by a Keyence BZ-9000 microscope. Five ovaries were used in each treatment group.

### Picrosirius red (PSR) staining

PSR staining was done to visualize the localization of collagen in ovary. After deparaffinization and washing with PBS (-), tissues were stained with a Picrosirius Red Stain Kit (Polysciences, PA, USA) according to the manufacturer’s recommendations. The tissues were observed under a Keyence BZ-9000 microscope (Keyence Co).

### TUNEL staining

The detection of apoptotic cells (containing fragmented DNA) in granulosa cells were performed by the TUNEL method (*In Situ* Cell Death Detection Kit POD; Roche Diagnostics) according to the manufacturer’s instructions. The nuclei were visualized with hematoxylin (Sakura-Finetek Japan). Digital images were captured using a Keyence BZ-9000 microscope.

### Quantitative measurement of AMH

The concentration of serum AMH at the diestrus stage was determined using mouse AMH ELISA Kit (E-EL-M0113, Elabscience Biotechnology, Bethesda, MD) according to the manufacturer’s instructions. These samples were analyzed using a microplate reader to determine the amount of substrate converted at 450 nm.

### Statistics

Statistical analyses of data from three or four replicates for comparison were carried out by either Student’s t-test or one-way ANOVA (Statview; Abacus Concepts, Inc., Berkeley, CA). All of data in this study were normally distributed.

## Supplementary information

Supplementary Information.
