# Supplementary material for: Pretreatment of ovaries with collagenase before vitrification keeps the ovarian reserve by maintaining cell-cell adhesion integrity in ovarian follicles
Source: Sci Rep. 2020 Apr 22;10:6841. doi: 10.1038/s41598-020-63948-y (PMC7176664; doi:10.1038/s41598-020-63948-y)
Supplement: Supplementary file 1 — Supplementary Information. [file 41598_2020_63948_MOESM1_ESM.pdf]

Supplemental information

Title: Pretreatment of ovaries with collagenase before vitrification keeps the ovarian reserve by maintaining cell-cell adhesion integrity in ovarian follicles

Running Title: cell adhesion in ovarian follicles during vitrification

Tomoko Kawai[1] and Masayuki Shimada[1]*

[1]Laboratory of Reproductive Endocrinology, Graduate School of Integrated Sciences for Life, Hiroshima University, Higashi-Hiroshima, Hiroshima, Japan

*Corresponding author: Masayuki Shimada, PhD, Laboratory of Reproductive Endocrinology, Graduate School of Integrated Sciences for Life, Hiroshima University, Higashi-Hiroshima, Hiroshima, 739-8528, Japan, Tel/Fax: (+81)-824-24-7899, E-mail: mashimad@hiroshima-u.ac.jp

Table S1. Primer list

Gene	Forward Primer	Reverse Primer	Size (bp)	Anneling temperature
Cdh1	5’-ACTGTGAAGGGACGGTCAAC-3’	5’-TGTCCCGGGTATCATCATCT-3’	192	60
Cdh2	5’-CCTGCTGATCCTTGTTCTCA-3’	5’-GGCATCAGGCTCCACAGTAT-3’	199	60
Cdh3	5’-GGCCCAGCTAACACATGACT-3’	5’-GGGGCAGTCATTGAACACTT-3’	208	60
Cdh4	5’-TTGGCCCCTATGTCTTTGAG-3’	5’-TATTGGACAAGGGAGGGTTG-3’	177	60
Cdh5	5’-CGGTCAAGTATGGGCAGTTT-3’	5’-GGTGAGGATGCAGAGGAAGA-3’	221	60
Cdh11	5’-AGGAGTATATGCCCGACGTG-3’	5’-AACAGCACAACGATGACCAG-3’	258	60
L19	5’-GGCATAGGGAAGAGGAAGG-3’	5’-GGATGTGCTCCATGAGGATGC-3’	199	60

Table S2. The antibody list

Target	The name of antibody	Catalog No.	Species
pan-cadherin	anti-pan cadherin monoclonal antibody	Biosensis (M1740-100)	mouse
connexin37	anti-Cx37/GJA4 polyclonal antibody	Abcam (181701)	rabbit
ZP3	anti-ZP3 polyclonal antibody	Proteintech (21279-1-AP)	rabbit
β-actin	anti-β actin monoclonal antibody	Sigma (A5316)	mouse

A

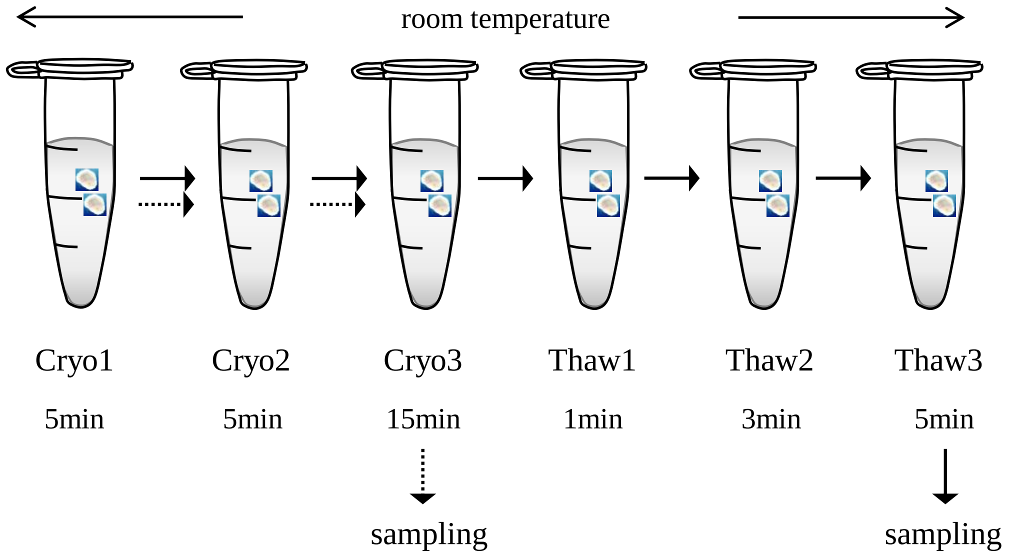

B

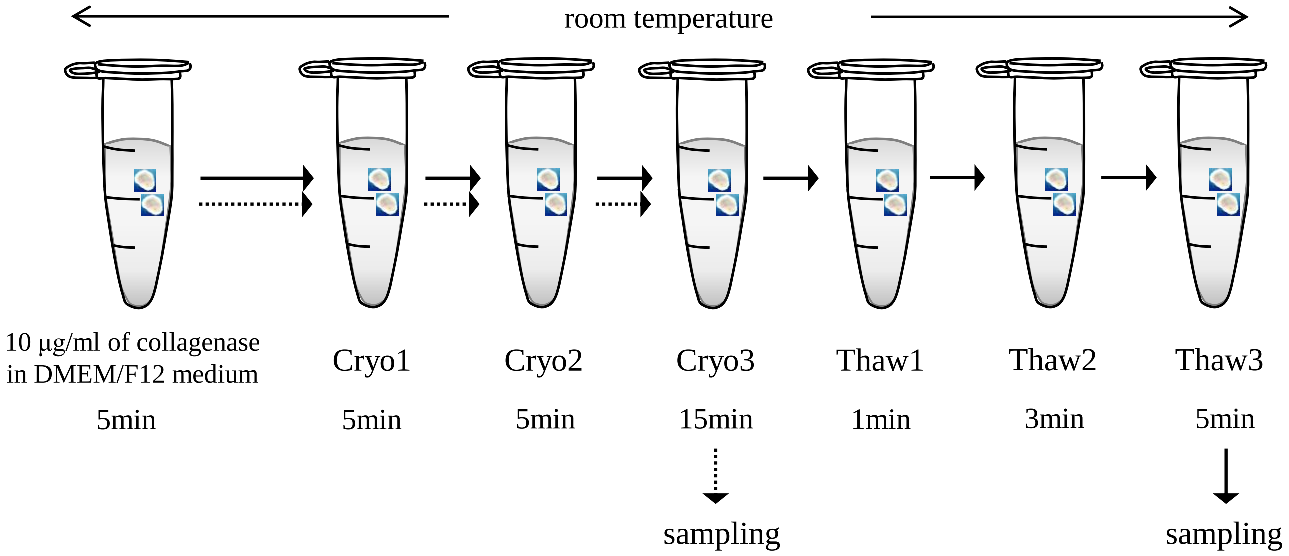

Supplemental figure 1

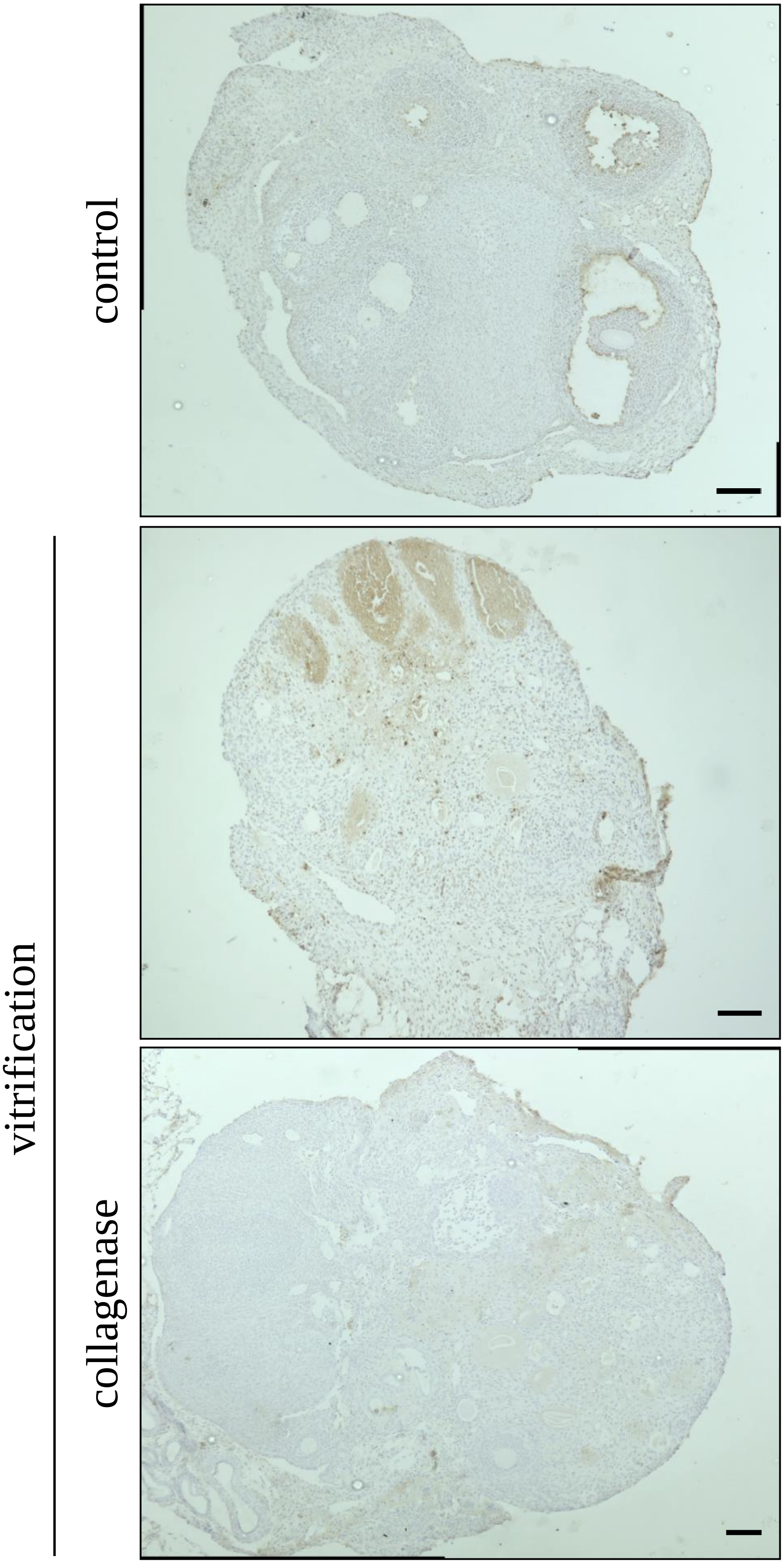

Supplemental figure 2

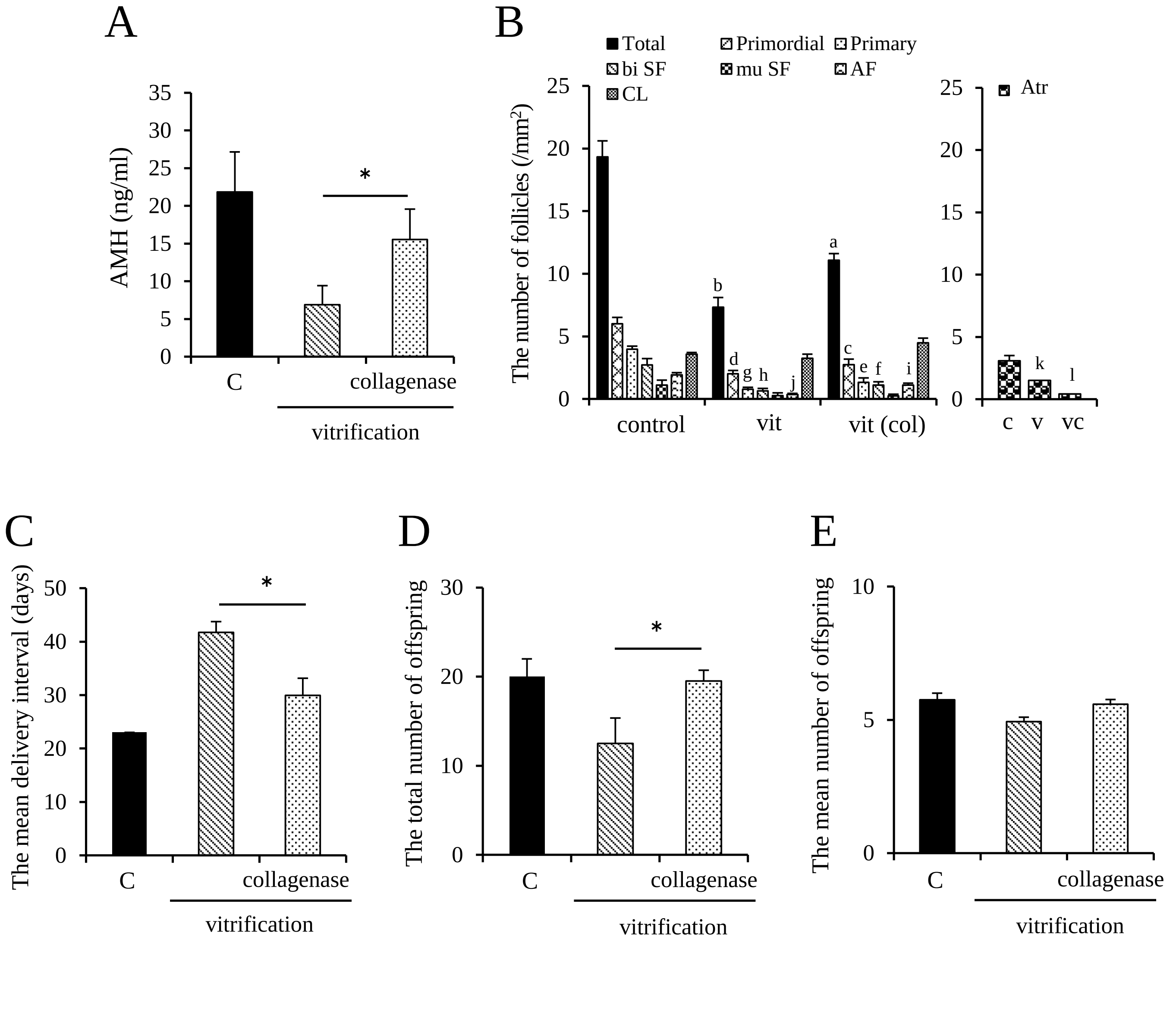

Supplemental figure 3

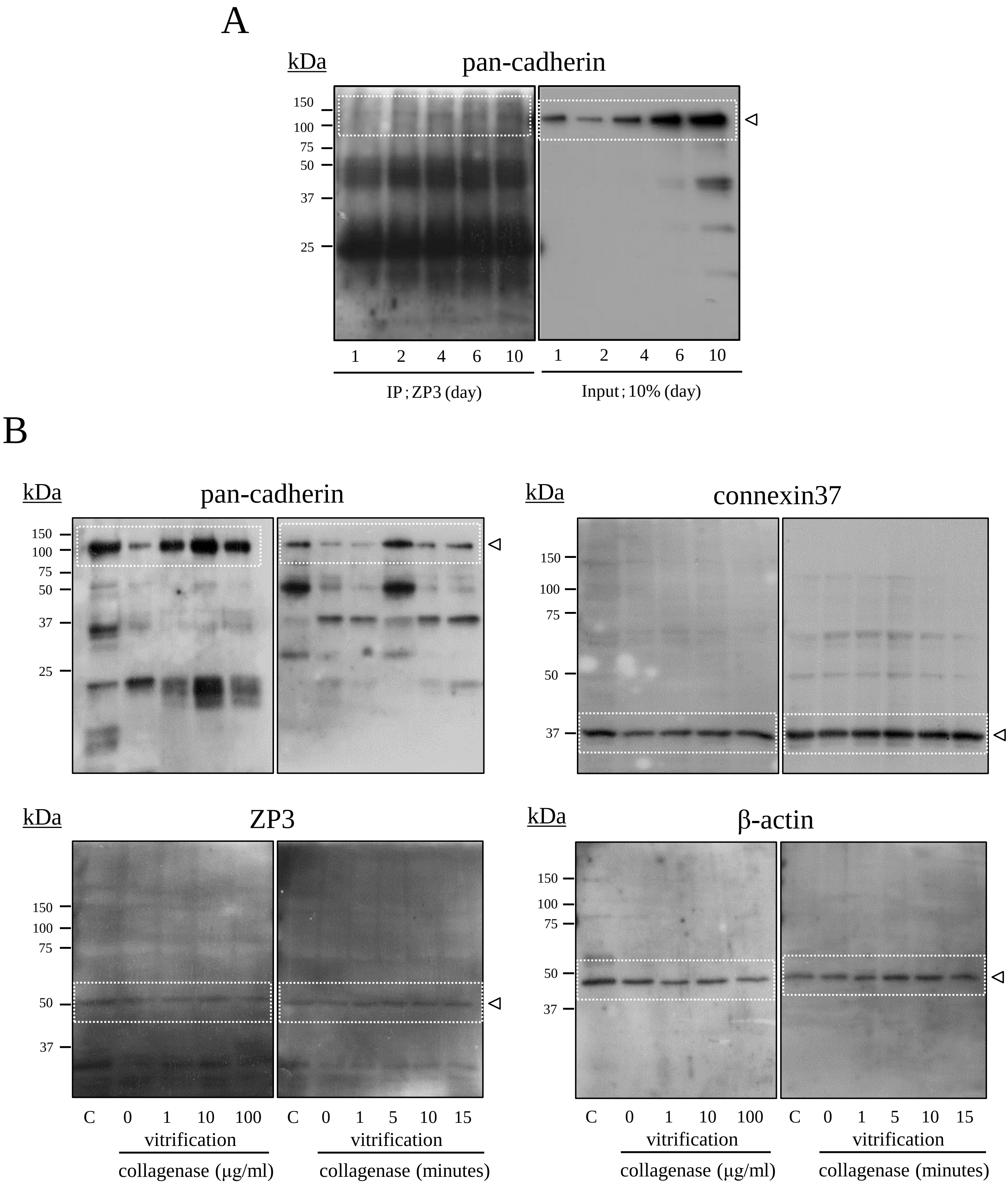

Supplemental figure 4
